# Diterpenoids from the Endophytic Fungus *Botryosphaeria* sp. P483 of the Chinese Herbal Medicine *Huperzia serrata*

**DOI:** 10.3390/molecules200916924

**Published:** 2015-09-17

**Authors:** Yan-Mei Chen, Yin-He Yang, Xiao-Nian Li, Cheng Zou, Pei-Ji Zhao

**Affiliations:** 1 State Key Laboratory of Phytochemistry and Plant Resources in West China, Kunming Institute of Botany, Chinese Academy of Sciences, Kunming 650201, China; E-Mails: ymchen2015@126.com (Y.-M.C.); yyh8612@126.com (Y.-H.Y.); lixiaonian@mail.kib.ac.cn (X.-N.L.); 2 School of Pharmaceutical Sciences, Kunming Medical University, Kunming 650500, China; E-Mail: zouchengkm@126.com

**Keywords:** *Botryosphaeria* sp. P483, tetranorlabdane diterpenoid, X-ray crystallography, antifungal activity, nematicidal activity

## Abstract

Two new tetranorlabdane diterpenoids, named botryosphaerins G (**1**) and H (**2**), were isolated from the solid fermentation products of *Botryosphaeria* sp. P483 along with seven known tetranorlabdane diterpenes (**3**–**9**). Their structures were elucidated by extensive analysis, including 1D and 2D nuclear magnetic resonance (NMR) spectroscopy, and high-resolution electrospray ionization mass spectrometry (HR-ESI-MS). Their absolute configuration was confirmed by single-crystal X-ray diffraction analyses using the anomalous scattering of Cu Kα radiation. All of the isolated compounds were tested for activity against phytopathogenic fungi and nematodes. Compounds **2** and **3** showed antifungal activity and compound **2** showed weak nematicidal activity.

## 1. Introduction

**Figure 1:**
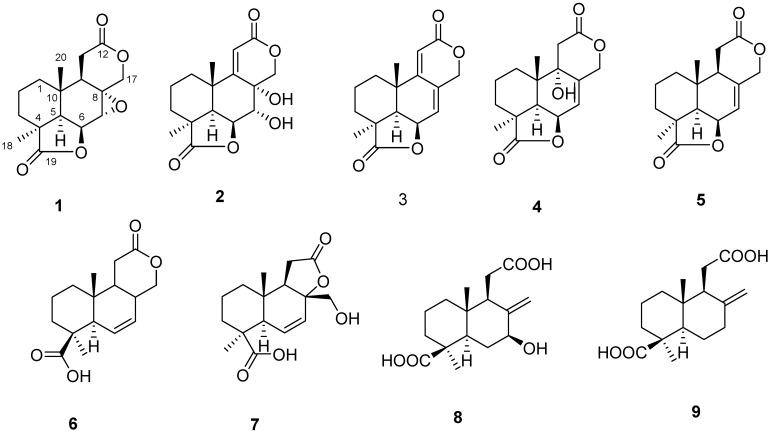
Chemical structures of compounds **1**–**9** from *Botryosphaeria* sp. P483.

Endophytic fungi, defined functionally by their occurrence in plant tissue without causing any overt effects [1], are present in almost all plants and are important sources of natural products [2]. The products of endophytic microbes and their uses in medicine, agriculture and industry have been reviewed [3]*.* The increasing number of new compounds discovered in endophytes demonstrates their potential for producing many more previously unknown natural products, which are still to be exploited for their potential applications. *Huperzia serrata* (Thunb.) Trev. (Huperziaceae) is a Chinese traditional medicine that produces huperzine A, a potential therapeutic agent for treatment of Alzheimer’s disease that has been extensively studied in recent years [4]. In our search for new active compounds from endophytic microorganisms, a series of novel compounds were previously identified [5,6,7]. During ongoing research on plant endophytic microorganisms, an isolate of *Botryosphaeria* sp. P483, obtained from the tissue of *H. serrata*, has been investigated. A systematic chemical study was performed and resulted in the isolation of new botryosphaerin G (**1**) and botryosphaerin H (**2**), along with seven known diterpenoids, 13,14,15,16-tetranorlabd-7-en-19,6β:12,17-diolide (**3**) [8], botryosphaerin A (**4**) [9], 3a,10b-dimethyl-1,2,3,3a,5a,7,10b,10c-octahydro-5,8-dioxa-acephenanthrylene-4,9-dione (**5**) [10], acrostalidic acid (**6**) [11], botryosphaerin B (**7**), LL-Z1271β (**8**) and acrostalic acid (**9**) [9] (Figure 1).

## 2. Results and Discussion

### 2.1. Characterization

The nucleotide sequences for the ITS1-5.8S rDNA-ITS4 region of the fungal strain P483 was registered in the GenBank database with the accession number KT213569, and the strain was determined to be *Botryosphaeria* sp. from the internal transcript spacer (ITS) analysis.

**Figure 2:**
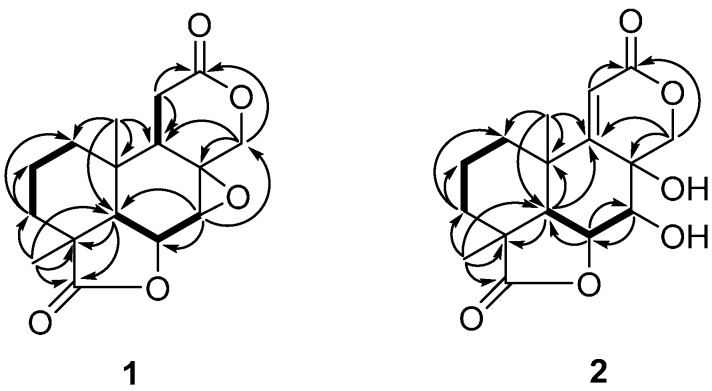
Key ^1^H-^1^H COSY (bold line) and HMBC (arrows) correlations for **1** and **2**.

**Figure 3:**
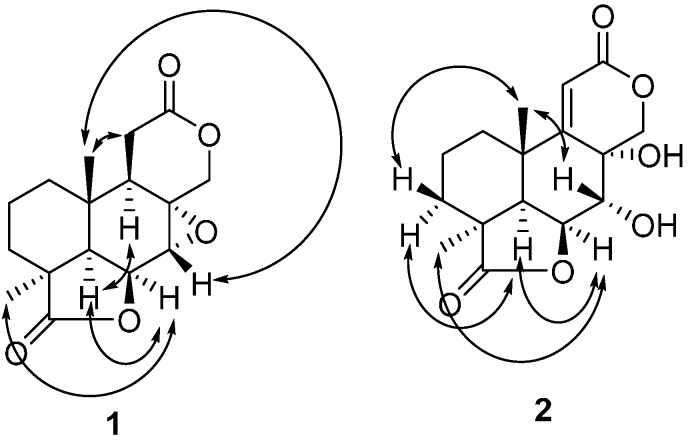
The NOESY correlations in **1** and **2**.

**Figure 4:**
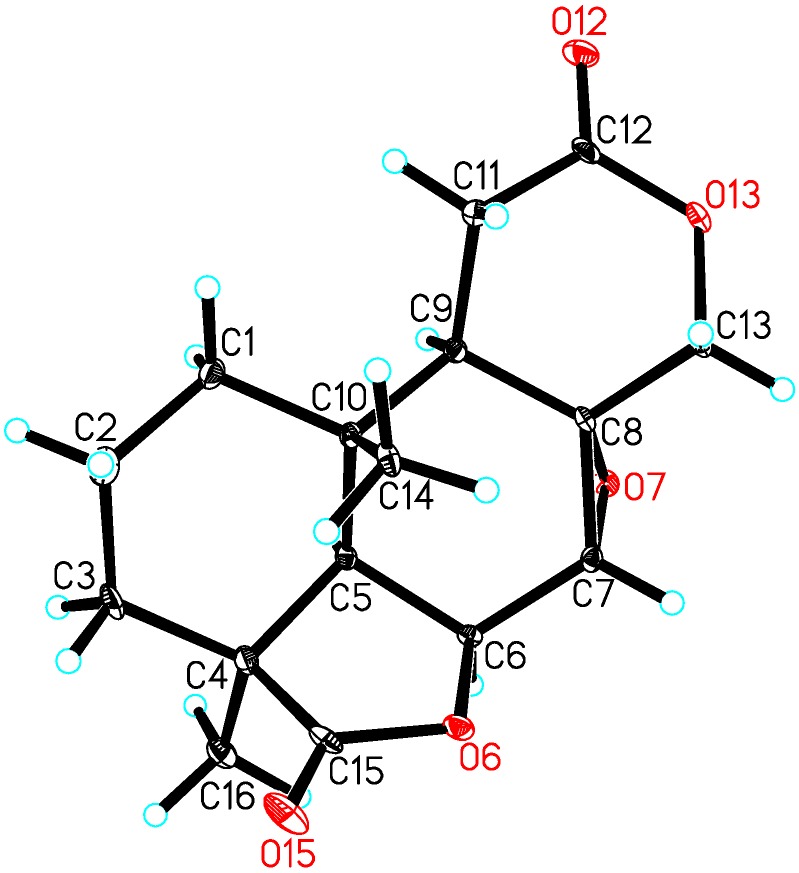
X-ray crystal structure of **1**.

Compound **1** was obtained as colorless needles. The negative (HR-ESI-MS) data indicated a molecular formula of C_16_H_20_O_5_ based on the [M + Na]^+^ ion signal at *m*/*z* 315.1203 (calc. 315.1208). The ^13^C-NMR and distortionless enhancement by polarization Transfer (DEPT) spectra (Table 1) revealed five quaternary carbons (δ_C_ 180.4, 171.6, 60.6, 41.7 and 34.0), four methines (δ_C_ 72.4, 53.6, 49.2 and 43.2), five methylenes (δ_C_ 71.2, 32.0, 28.51, 28.45, and 17.5) and two methyls (δ_C_ 24.4 and 17.6). According to the ^1^H-NMR (Table 1), two singlet methyl signals (δ_H_ 1.28 and 0.93) were also present, which suggested that compound **1** was a tetranorlabdane diterpene [9,10,11]. In the COSY spectrum (Figure 2 and Figure S5), three fragments were deduced to be –C-1–C-2–C-3–, –C-5–C-6–C-7– and –C-9–C-11–from a complete interpretation of the key correlations. The HMBC experiment (Figure 2 and Figure S4) showed that the methyl protons at δ_H_ 0.93 (H-20) correlated with the carbons at δ_C_ 49.2 (C-5), 43.2 (C-9), 34.0 (C-10) and 32.0 (C-1); the protons at δ_H_ 2.21 and 1.49 (H-3) correlated with carbons at δ_C_ 180.4 (C-19), 49.2 (C-5), 41.7 (C-4), 32.0 (C-1), 24.4 (C-18) and 17.6 (C-20); the proton at δ_H_ 4.90 (H-6) correlated with carbons at δ_C_ 60.6 (C-8), 53.6 (C-7) and 34.0 (C-10); the methyl protons at δ_H_ 1.28 (H-18) correlated with carbons at δ_C_ 180.4 (C-19), 49.2 (C-5), 41.7 (C-4) and 28.51 (C-3); the protons of the oxygenated methylene at δ_H_ 4.41 and 4.05 (H-17) correlated with carbons at δ_C_ 171.6 (C-12), 60.6 (C-8), 53.6 (C-7) and 43.2 (C-9). These data, together with other correlations (Figure 2), established the planar structure. The NOESY experiment showed NOEs between H-6 and H-18, H-5; H-5 and H-9; H-20 and H-7, H-11 (Figure 3 and Figure S6). These data supported the relative configurations of C-5, C-6, C-7, C-9, C-18 and C-20. The final refinement of the Cu Kα data resulted in a Flack parameter = 0.2(3) and a Hooft parameter = 0.07(6) for 1024 Bijvoet pairs [12,13], allowing an explicit assignment of the complete absolute configuration of **1** as shown in Figure 1 with 4*S*, 5*R*, 6*S*, 7*R*, 8*R*, 9*R* and 10*S* stereochemistry, which was named as botryosphaerin G (Figure 4).

Compound **2** was obtained as a colorless solid. The positive HR-ESI-MS data indicated a molecular formula of C_16_H_20_O_6_ based on the [M + H]^+^ at *m*/*z* 309.1331 (calc. 309.1338), and this was supported by the ^13^C-NMR and DEPT data (Table 1).

**Table 1:** NMR data of compounds **1** and **2**.

Position	1 ^a^			2 ^b^		
	δ_H_ (multi, *J* in Hz)	δ_C_	HMBC	δ_H_ (multi, *J* in Hz)	δ_C_	HMBC
1β	1.54, m	32.0	3, 10	1.76, m	33.9	2, 3, 5, 9, 10, 20
1α	1.15, m		3, 9, 10	1.48, m		2, 3, 10, 20
2β	1.65, m	17.5	1, 3, 4	1.60, m	17.7	2, 4, 10
2α	1.59, m		1, 3, 4	1.43, m		1, 3
3β	2.21, dt, 14.4, 5.3	28.51	1, 4, 5, 18, 19, 20	2.21, m	27.8	1, 2, 4, 5, 18, 19
3α	1.49, m		1, 4, 5, 18, 19, 20	1.35, m		1, 2, 4, 5, 18, 19
4	-	41.7	-	-	42.1	-
5	1.67, d, 4.7	49.2	1, 4, 10, 18, 19, 20	2.86, d, 5.5	47.0	1, 3, 4, 9, 10, 18, 19, 20
6	4.90, d, 4.7	72.4	7, 8, 10	5.40, t, 5.5	84.0	4, 7, 10
7	3.56, brs	53.6	5, 6, 17	4.73, d, 5.5	71.8	6, 17
8	-	60.6	-	-	68.5	-
9	1.90, dd, 6.4, 11.4	43.2	5, 8, 10, 11, 12, 20	-	168.0	-
10	-	34.0	-	-	35.7	-
11α	2.70, dd, 6.4, 15.2	28.45	8, 9, 10, 12	6.11, s	114.9	8, 9, 10, 12, 20
11β	2.54, dd, 11.4, 15.2		8, 9, 10, 12			
12	-	171.6	-	-	164.7	-
17β	4.41, d, 12.8	71.2	7, 8, 9, 12	5.18, d, 11.9	75.7	8, 9, 10
17α	4.05, d, 12.8		7, 8, 9, 12	4.62, d, 11.9		8
18	1.28, s	24.4	3, 4, 5, 19	1.19, s	24.4	4, 5, 19, 20
19	-	180.4	-	-	182.2	-
20	0.93, s	17.6	1, 5, 9, 10,	1.20, s	27.7	1, 5, 9, 10

^a^ Recorded in CDCl_3_. The ^1^H- and ^13^C- spectra were recorded at 400 MHz and the 2D-NMR at 600 MHz; ^b^ Recorded in C_5_D_5_N. The ^1^H- and ^13^C- spectra were recorded at 400 MHz and the 2D-NMR at 600 MHz.

Compound **2** was very similar to compound **1**, but the epoxide ring of **1** was replaced by two hydroxyls and there was an additional double bond (Figure 1). The HMBC experiment (Figure 2 and Figure S10) showed that the olefinic proton at δ_H_ 6.11 (H-11) correlated with the carbons at δ_C_ 168.0 (C-9), 164.7 (C-12), 68.5 (C-8), 35.7 (C-10) and 27.7 (C-20); the proton of the oxygenated methine at δ_H_ 5.40 (H-18) correlated with carbons at δ_C_ 71.8 (C-7), 47.0 (C-5) and 35.7 (C-10); the protons at δ_H_ 4.73 (H-7) correlated with carbons at δ_C_ 84.0 (C-6) and 75.7 (C-17). These data, together with other correlations (Figure 2), established the planar structure. The NOESY experiment showed NOE interactions between H-6 and H-18, H-5; between H-20 and H-7, H-3β; and between H-5 and H-3α (Figure 3 and Figure S12). From comparison of the NMR data and the specific rotation data of compounds **1** and **2**, together with a biogenetic perspective, the absolute configuration of **2** is proposed to be 4*S*, 5*R*, 6*S*, 7*R*, 8*R*, 9*Z*, and 10*S*. Compound **2** was named as botryosphaerin H.

### 2.2. Biological Activities

Compounds **1**–**9** were assayed for antifungal activity against *Gaeumannomyces graminis*, *Fusarium moniliforme*, *Fusarium solani*, *Fusarium oxysporum* and *Pyricularia oryzae*. Compounds **2** and **3** showed strong antifungal activity at 100 μg/disk (Table 2). When assayed for nematicidal activity against *Panagrellus redivivus* and *Caenorhabditis* *elegans*, only botryosphaerin H (**2**) was active. Compound **2** killed 30% of *P*. *redivivus* and 28% of *C.* *elegans* at 400 mg·L^−1^ at 24 h, while the control (5% acetone) killed only 1.5% at 24 h. As a positive control, avermectin was used and it killed 35% of *P*. *redivivus* and 92% of *C.* *elegans* at 400 mg·L^−1^ at 24 h.

**Table 2:** Antifungal activity of compounds **2** and **3** from *Botryosphaeria* sp. P483 at 100 μg/disk.

Compounds	Diameter of Fungus-Free Zone (mm)				
	*G. graminis*	*F. solani*	*P. oryzae*	*F. moniliforme*	*F. oxysporum*
**2**	9	7	7	8	8
**3**	12	10	10	11	13
Carbendazim (50 μg/disk)	14	18	15	17	15
Control (methanol)	0	0	0	0	0

## 3. Experimental Section

### 3.1. General

The optical rotations were measured using a Jasco DIP-370 digital polarimeter (Tokyo, Japan). The UV spectra were recorded on a Shimadzu UV-2401PC spectrophotometer (Tokyo, Japan). The NMR spectra were obtained on Bruker AM-400 and Avance III 600 spectrometers (Karlsruhe, Germany). The ESI and HR-ESI-MS were recorded on Finnigan LCQ-Advantage (San Jose, CA, USA) and VG Auto-Spec-3000 mass spectrometers (Manchester, UK), and EI-MS was recorded on a Waters AutoSpec Premier P776 (Millford, MA, USA). Column chromatography was performed on silica gel G, silica gel 254, silica gel 200–300 mesh (Qingdao Marine Chemical Factory, Qingdao, China), silica gel H (Merck, Darmstadt, Germany) and Sephadex LH-20 (Amersham Pharmacia, Uppsala, Sweden).

### 3.2. Fungal Material

The plant, *Huperzia serrata* (Thunb.) Trev., was collected in Xichou County, Yunnan Province, China, in July 2013. A voucher specimen (No. 20130710ZPJ) was deposited at the Herbarium of Kunming Institute of Botany (KUN), Chinese Academy of Sciences. The plant materials were washed under running tap water, sterilized successively with 75% ethanol for 1 min and 15% sodium hypochlorite for 15 min, then rinsed in sterile water five times and cut into small pieces. These small pieces were incubated at 28 °C on PDA media (potato 200 g, dextrose 20 g, agar 15 g, distilled water 1 L) and cultured until a colony or mycelium appeared around the segments. A strain, designated as F483, appeared after culturing for about two weeks and was isolated from the sterilized branch. The material was deposited in Kunming Institute of Botany, Chinese Academy of Sciences, Kunming, China.

### 3.3. Fermentation, Extraction and Isolation

*Botryosphaeria* sp. 483 was cultured on PDA at 28 °C for 16 days. The solid culture (30 L, about 1000 Petri dishes) products were cut into small pieces and extracted three times with EtOAc/MeOH/AcOH (80:15:5, *v*/*v*/*v*). The combined extracts were evaporated, then, the residue was suspended in water and extracted three times with EtOAc. The combined ethyl acetate extracts were evaporated and the residue (27 g) was subjected to column chromatography on silica gel G (200–300 mesh, 6.5 × 45 cm, 200 g) eluted with a gradient of petroleum ether–EtOAc (from 10:1 to 6:4) followed by a gradient of CHCl_3_–MeOH (from 20:1, 10:1, 9:1, 8:2, 7:3, MeOH, each 1.5 L, 15 mL/min) from which 12 fractions were collected (Fr.1–Fr.12). Further chromatography of Fr.5 (705 mg) on Sephadex LH-20 (30 g, 2.5 × 120 cm, 1 mL/min) eluted with CHCl_3_–MeOH (1:1, about 500 mL) was conducted and four fractions were collected (Fr.5.1–Fr.5.4). Chromatography of Fr.5.4 (185 mg) on a column of silica gel (GF254, 3.5 × 43 cm, 15 g) eluted with petroleum ether–acetone (10:1) followed by purification on Sephadex LH-20 (30 g, 2.5 × 120 cm, 1 mL/min) eluted with MeOH (about 500 mL) gave compound **9** (3.1 mg).

Chromatography of Fr.6 (1.8 g) on Sephadex LH-20 (40 g, 2.5 × 150 cm, 1 mL/min) eluted with CHCl_3_–MeOH (1:1, about 1000 mL) was collected in four fractions (Fr.6.1–Fr.6.4). Purification of Fr.6.2 (480 mg) on a column of silica gel (GF254, 3.5 × 43 cm, 20 g) eluted with CHCl_3_–acetone (200:1, 800 mL) gave compound **1** (13.4 mg). Chromatography of Fr.6.3 (600 mg) on silica gel (GF254, 3.5 × 43 cm, 20 g) eluted with a gradient of CHCl_3_–acetone (from 200:1, 100:2, 100:6, each 500 mL, 1 mL/min) gave compound **6** (6.4 mg).

Chromatography of Fr.7 (1.5 g) was subjected on Sephadex LH-20 (40 g, 2.5×150 cm, 1 mL/min) eluted with CHCl_3_–MeOH (1:1, about 800 mL), and then separated on silica gel (GF254, 3.5 × 60 cm, 40 g) eluted with petroleum ether-acetone (from 100:1, 50:1, 20:1, 10:1, each 200 mL) to give, in three fractions, (Fr.7.1–Fr.7.3). Purification of Fr.7.2 (95 mg) on Sephadex LH-20 (30 g, 2.5×120 cm, 1 mL/min) eluted with MeOH (about 400 mL) gave compound **7** (11.5 mg).

Chromatography of Fr.8 (4.8 g) was conducted on Sephadex LH-20 (40 g, 2.5 × 150 cm, 1 mL/min) eluted with CHCl_3_–MeOH (1:1, about 1000 mL) and two fractions were collected (Fr.8.1, Fr.8.2). Purification of Fr.8.1 (1.2 g) on silica gel (GF254, 3.5 × 60 cm, 40 g) eluted with petroleum ether-acetone (4:1, 2000 mL) gave compound **5** (16.1 mg). Chromatography of Fr.8.2 (2.4 g) on silica gel (GF254, 3.5 × 80 cm, 60 g) eluted with a gradient of petroleum ether–acetone (100:7, 9:1, 4:1, each 600 mL) was followed by purification on silica gel (GF254, 3.5 × 43 cm, 10 g) eluted with CHCl_3_–MeOH (100:8, about 400 mL) to give compound **8** (13.4 mg).

Further chromatography was conducted with Fr.9 (1.5 g) on silica gel (GF254, 3.5 × 60 cm, 40 g) eluted with a gradient of CHCl_3_–acetone (100:1, 20:1, 10:1, 9:1, 4:1 each 300 mL) and seven fractions were collected (Fr.9.1–Fr.9.7). Purification of Fr.9.3 (80 mg) on silica gel (GF254, 3.5 × 43 cm, 10 g) eluted with petroleum ether-acetone (8:1, about 400 mL) gave compound **3** (30.4 mg). Purification of Fr.9.5 (600 mg) on silica gel (GF254, 3.5 × 43 cm, 15 g) eluted with CHCl_3_–acetone (50:1, about 600 mL) gave compound **2** (4.3 mg) and chromatography of Fr.9.6 (213 mg) on Sephadex LH-20 (30 g, 2.5 × 120 cm, 1 mL/min) eluted with MeOH (about 500 mL) gave compound **4** (20.4 mg).

*Botryosphaerin G* (**1**): C_16_H_20_O_5,_ colorless needles; mp 225–226 °C ${\text{[α]}}_{D}^{21}$ = +63.0 (*c* = 0.16, MeOH); UV (MeOH) λ_max_ (log ε): 200 (2.73), 218 (2.70), 264 (1.96); ESI-MS: 293 [M + H]^+^; HR-ESI-MS ([M + Na]^+^ *m*/*z* 315.1203; calc. 315.1208). ^1^H-, ^13^C- and HMBC NMR see Table 1.

Crystal data: C_16_H_20_O_5_, *M*_r_ = 292.32, orthorhombic, *a* = 9.6701(3) Å, *b =* 9.8719(3) Å, *c* = 14.2941(4) Å, α = 90.00°, β = 90.00°, γ = 90.00°, *V* = 1364.55(7) Å^3^, *T* = 100(2) K, space group *P*2_1_2_1_2_1_, *Z* = 4, μ(CuKα) = 0.871 mm^−1^, 9249 reflections measured, 2490 independent reflections (*R_int_* = 0.0547). The final *R*_1_ values were 0.0604 (*I* > 2σ(*I*)). The final *wR*(*F*^2^) values were 0.1513 (*I* > 2σ(*I*)). The final *R*_1_ values were 0.0607 (all data). The final *wR*(*F*^2^) values were 0.1517 (all data). The goodness of fit on *F*^2^ was 1.054. Flack parameter = 0.2(3). The Hooft parameter was 0.07(6) for 1024 Bijvoet pairs. Crystallographic data (excluding structure factors) have been deposited at the Cambridge Crystallographic Data Centre under the reference number CCDC 1411361. Copies of the data can be obtained free of charge on application to the CCDC, 12 Union Road, Cambridge CB2 IEZ, UK. Fax: +44-(0)1223-336033 or e-mail: deposit@ccdc.cam.ac.uk.

*Botryosphaerin H* (**2**): C_16_H_20_O_6_, colorless amorphous solid; ${\text{[α]}}_{D}^{21}$ = +38.0 (*c* = 0.17, MeOH); UV (MeOH) λ_max_ (log ε): 216 (3.96); ESI-MS: 309 [M + H]^+^; HR-ESI-MS ([M + H]^+^ *m*/*z* 309.1331; calc. 309.1338). ^1^H-, ^13^C- and HMBC NMR see Table 1.

### 3.4. Bioassays

Antifungal activity was assayed against phytopathogenic fungi (*G.* *graminis*, *F.* *moniliforme*, *F.* *solani,* *F.* *oxysporum* and *P.* *oryzae*) using the disk diffusion method [14], and carbendazim was as a positive control in antifungal activity. Determination of nematicidal activity against *P. redivivus* and *C. elegans* was based on the literature method [15]. Avermectin was as a positive control in nematicidal activity (Lynhi Fine Chemical Co. Ltd, Shijiazhuang, China).

## 4. Conclusions

A endophytic fungus, *Botryosphaeria* sp. P483, isolated from the Chinese Herbal Medicine *Huperzia serrata*, has been investigated. A systematic chemical study was performed and resulted in the isolation of new botryosphaerin G (**1**) and botryosphaerin H (**2**), along with seven known diterpenoids, 13,14,15,16-tetranorlabd-7-en-19,6β:12,17-diolide (**3**), botryosphaerin A (**4**), 3a,10b-dimethyl-1,2,3,3a,5a,7,10b,10c-octahydro-5,8-dioxa-acephenanthrylene-4,9-dione (**5**), acrostalidic acid (**6**), botryosphaerin B, LL-Z1271β (**8**) and acrostalic acid (**9**). Compounds **2** and **3** showed antifungal activity and compound **2** showed weak nematicidal activity.

## Supplementary Materials

Supplementary materials can be accessed at: http://www.mdpi.com/1420-3049/20/09/16924/s1.
