# Supplementary material for: Diterpenoids from the Endophytic Fungus Botryosphaeria sp. P483 of the Chinese Herbal Medicine Huperzia serrata
Source: Molecules. 2015 Sep 17;20(9):16924–32. doi: 10.3390/molecules200916924 (PMC6331882; doi:10.3390/molecules200916924)
Supplement: Supplementary file 1 [file molecules-20-16924-s001.pdf]

Supplementary Information

Figures S1–S6 The ^{1}H-, ^{13}C-NMR, HSQC, HMBC, COSY and ROESY of botryosphaerin G in $CHCl_3$.

Figures S7–S12 The ^{1}H-, ^{13}C-NMR, HSQC, HMBC, COSY and ROESY of botryosphaerin H in C_5D_5N.

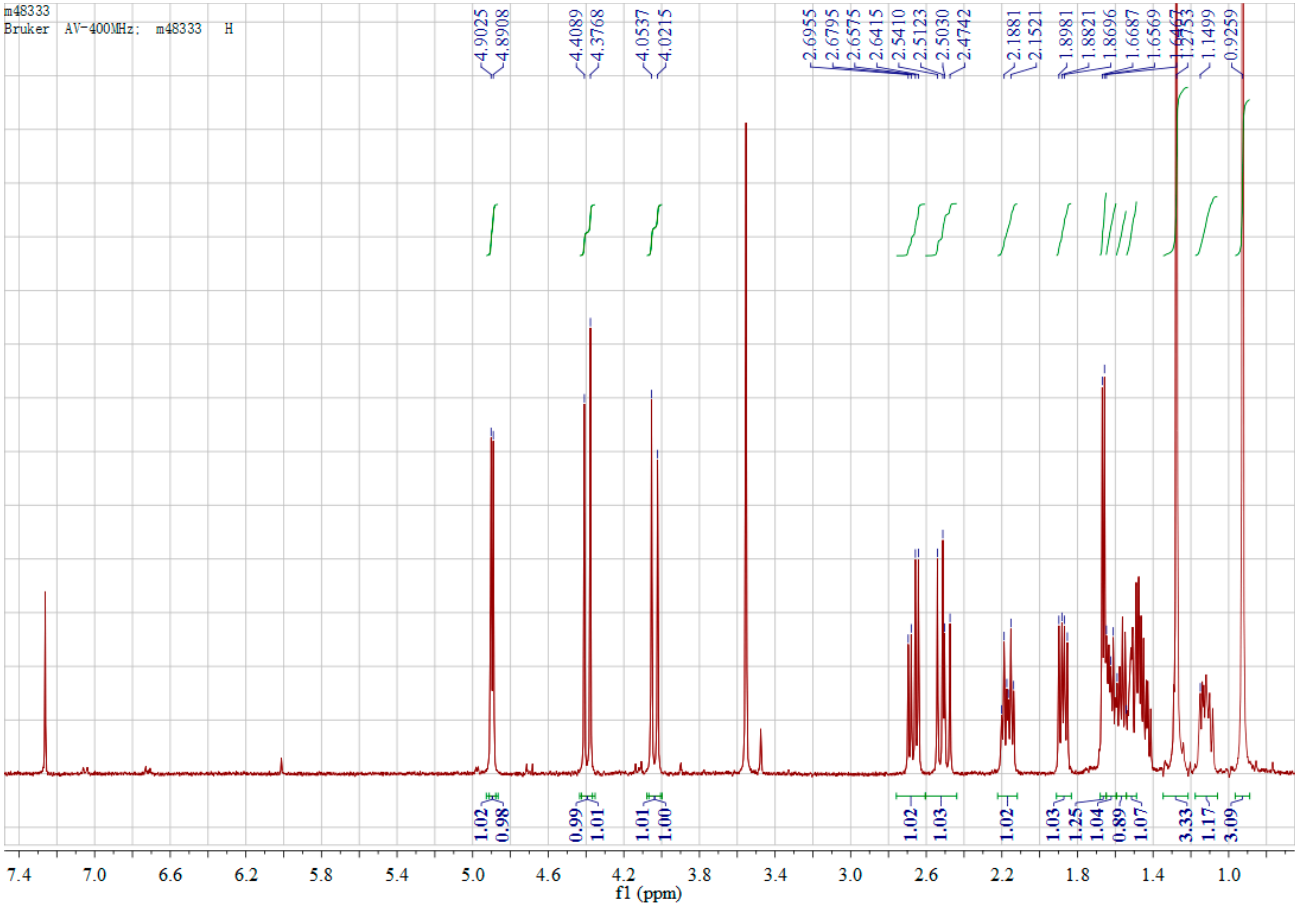

Figure S1. The ^{1}H-NMR of botryosphaerin G in $CHCl_3$.

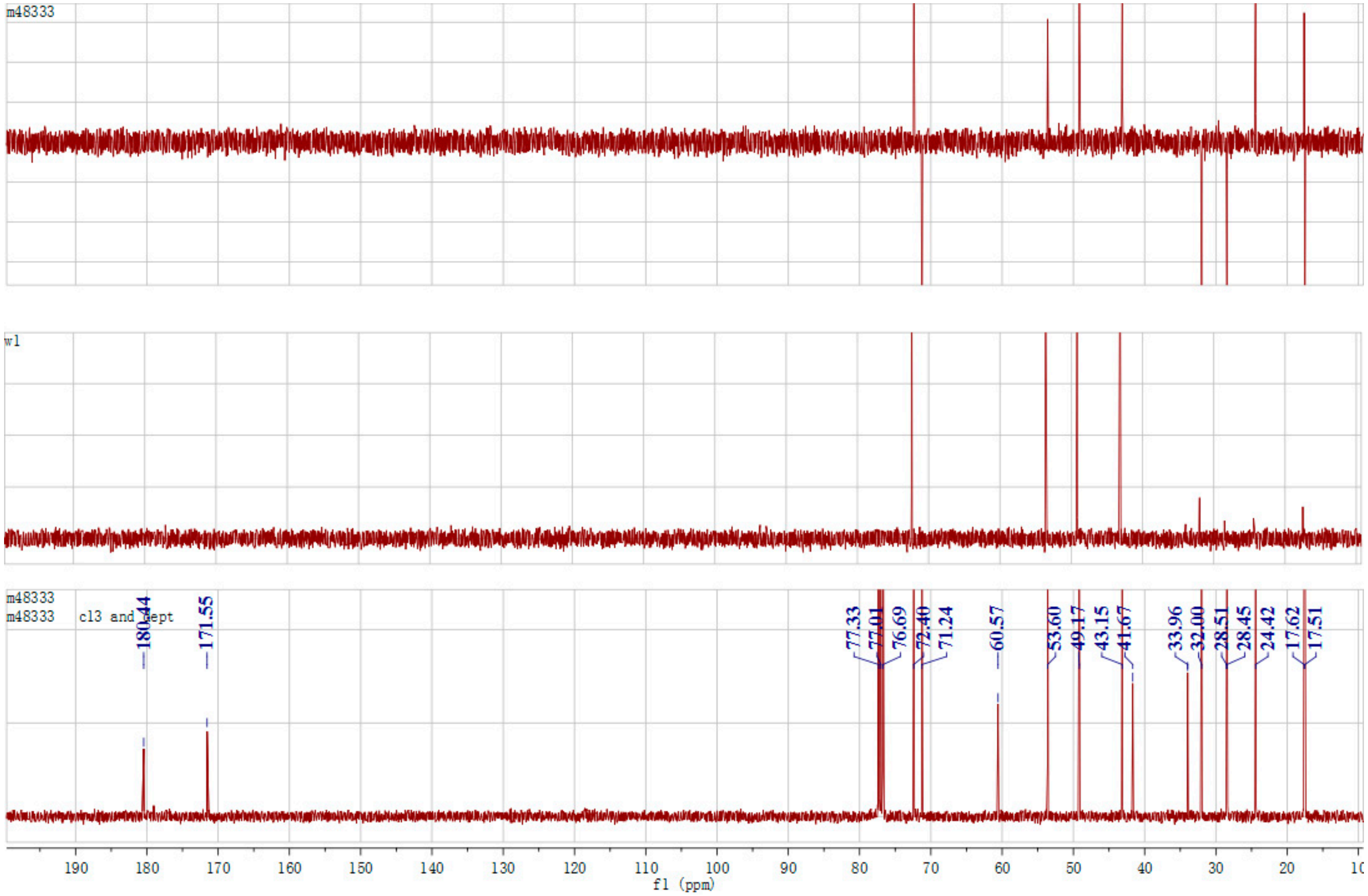

Figure S2. The ^{13}C-NMR of botryosphaerin G in $CHCl_3$.

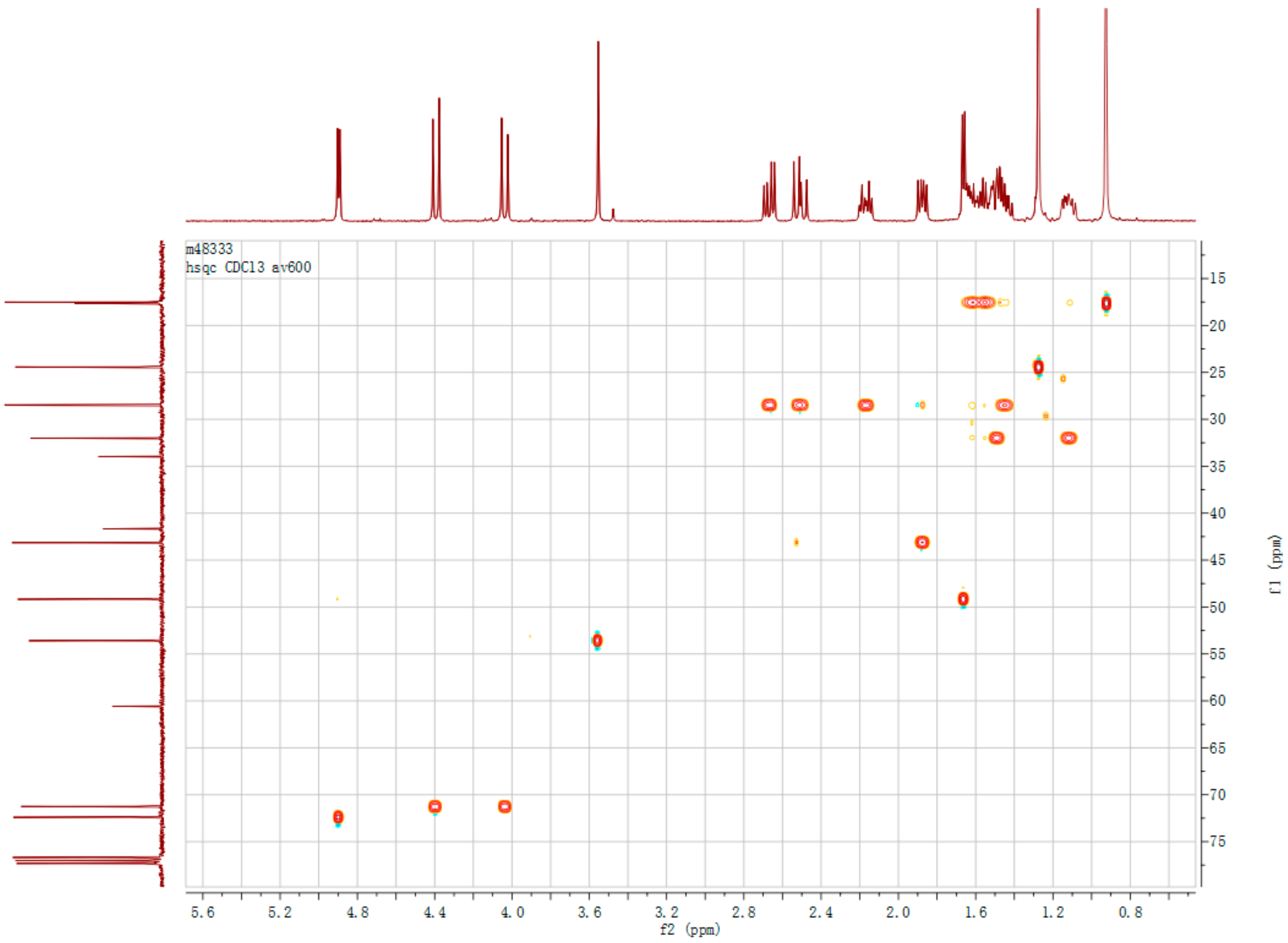

Figure S3. The HSQC NMR of botryosphaerin G in $CHCl_3$.

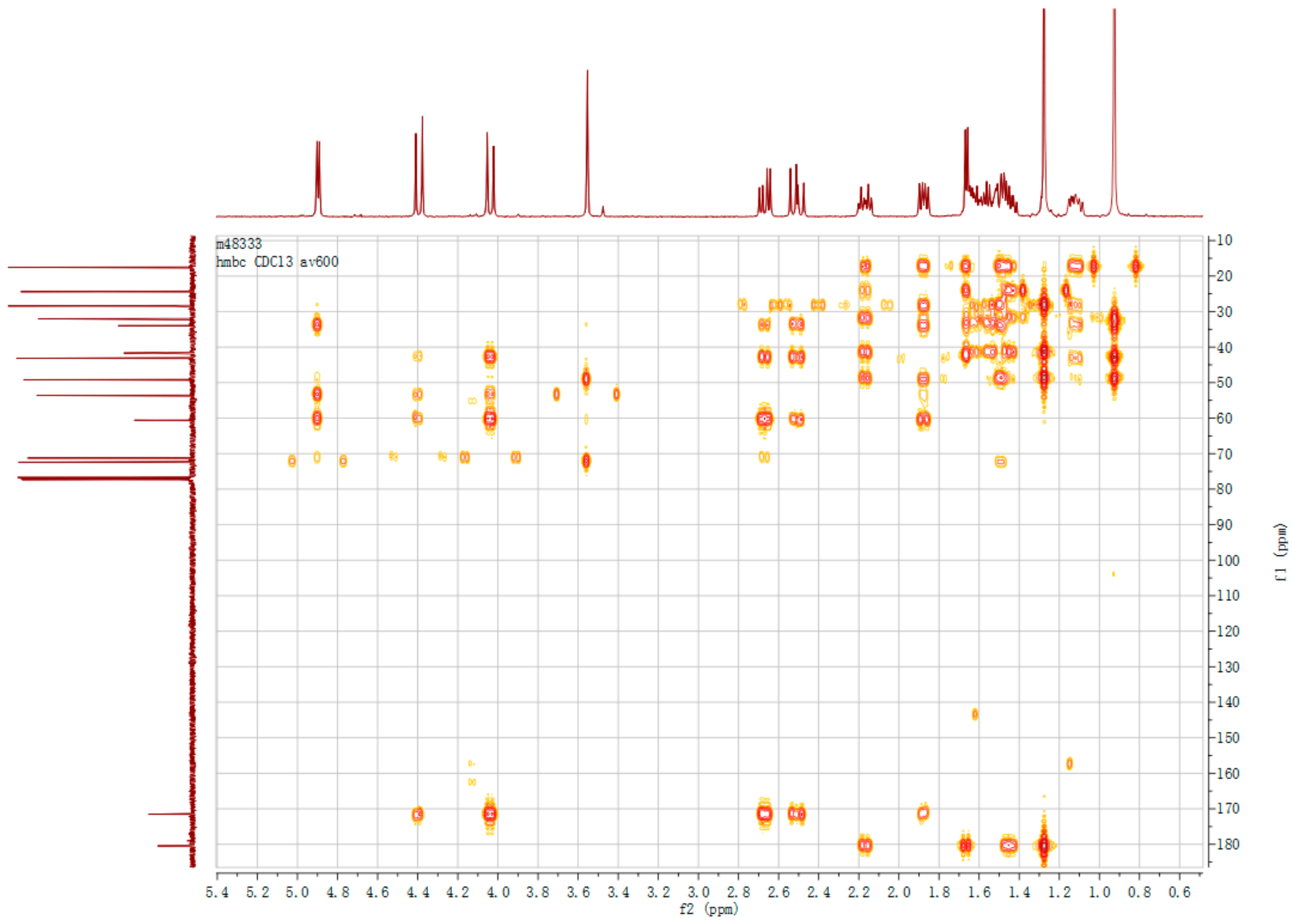

Figure S4. The HMBC NMR of botryosphaerin G in $CHCl_3$.

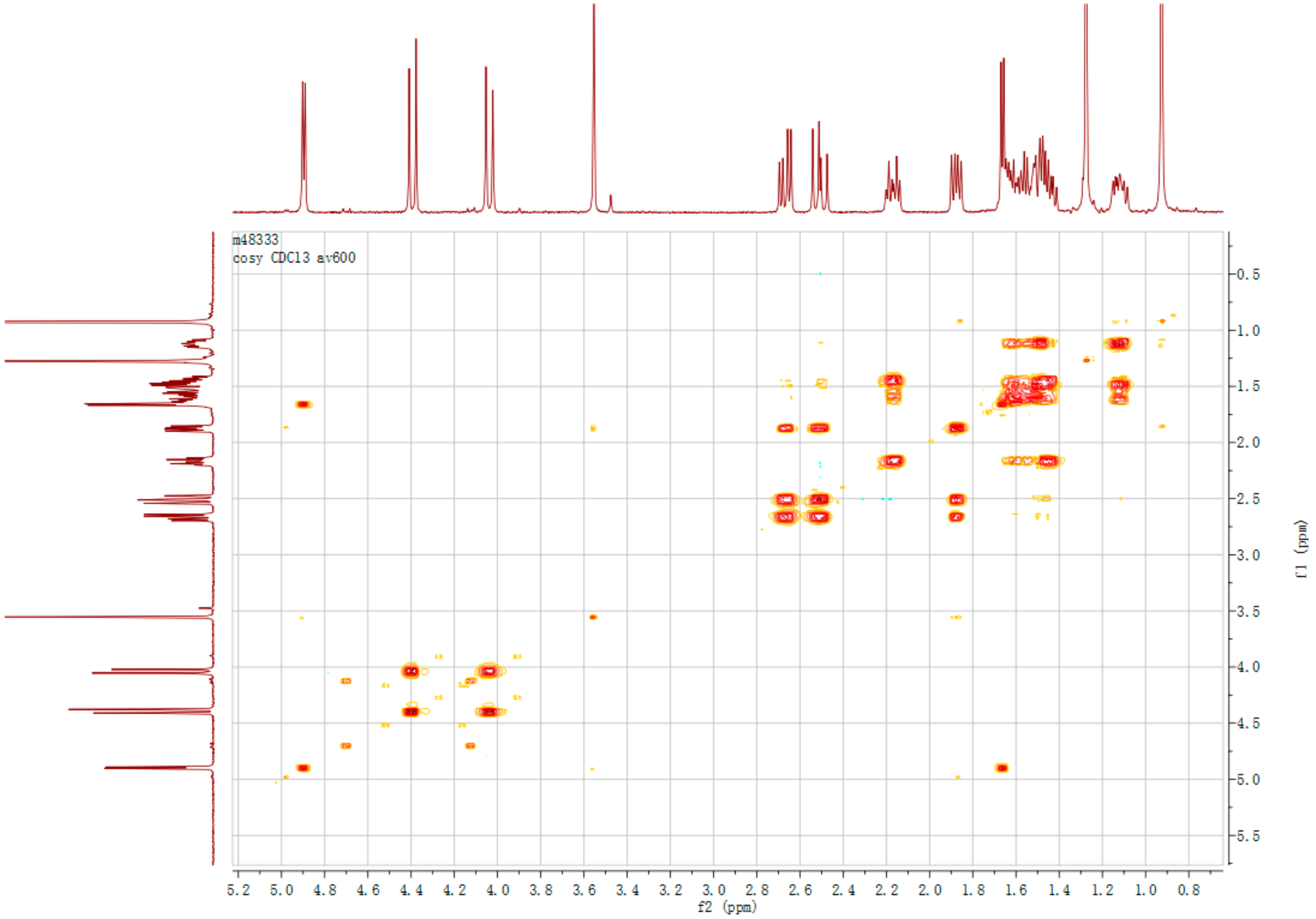

Figure S5. The COSY NMR of botryosphaerin G in $CHCl_3$.

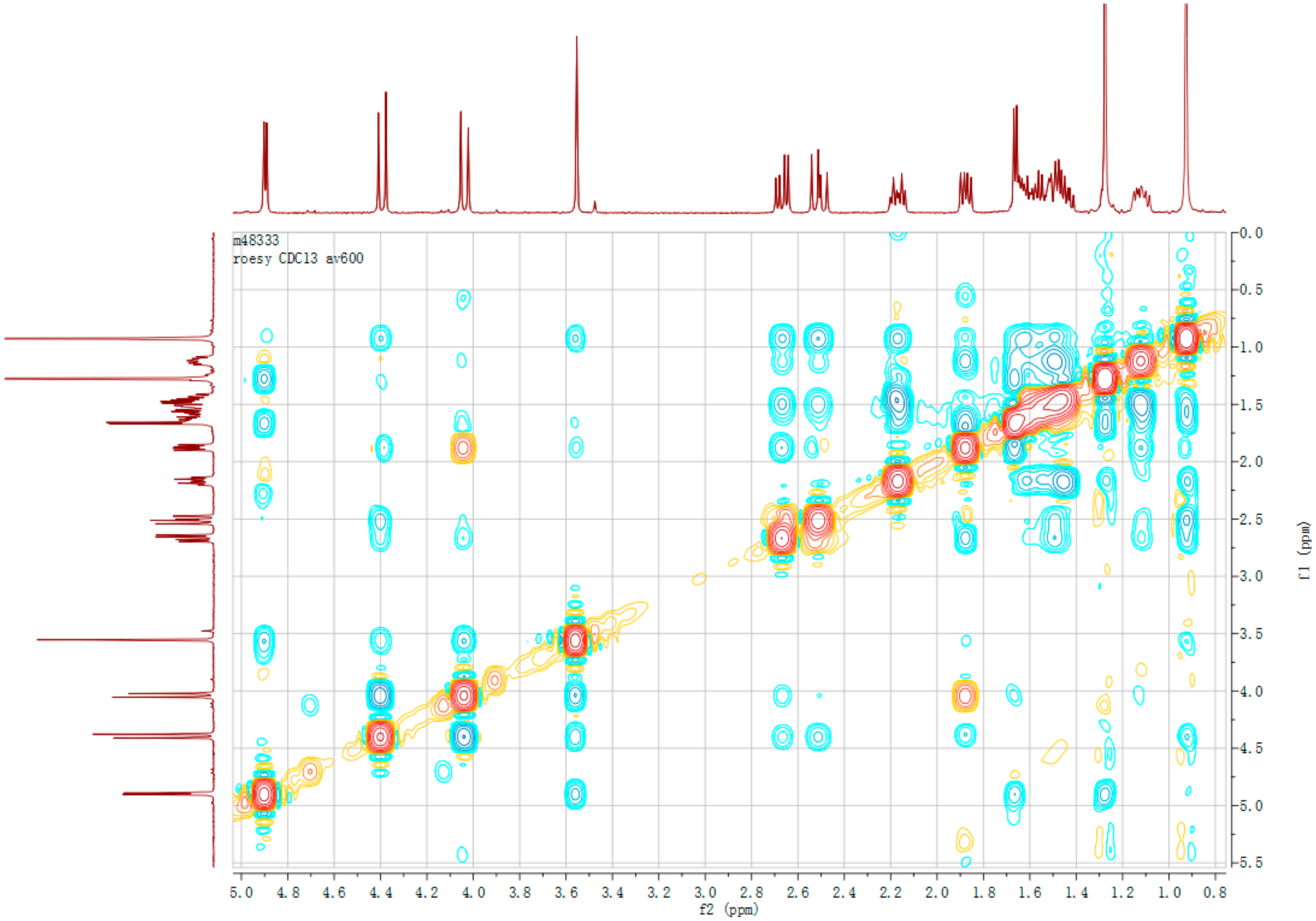

Figure S6. The ROESY NMR of botryosphaerin G in CHCl3.

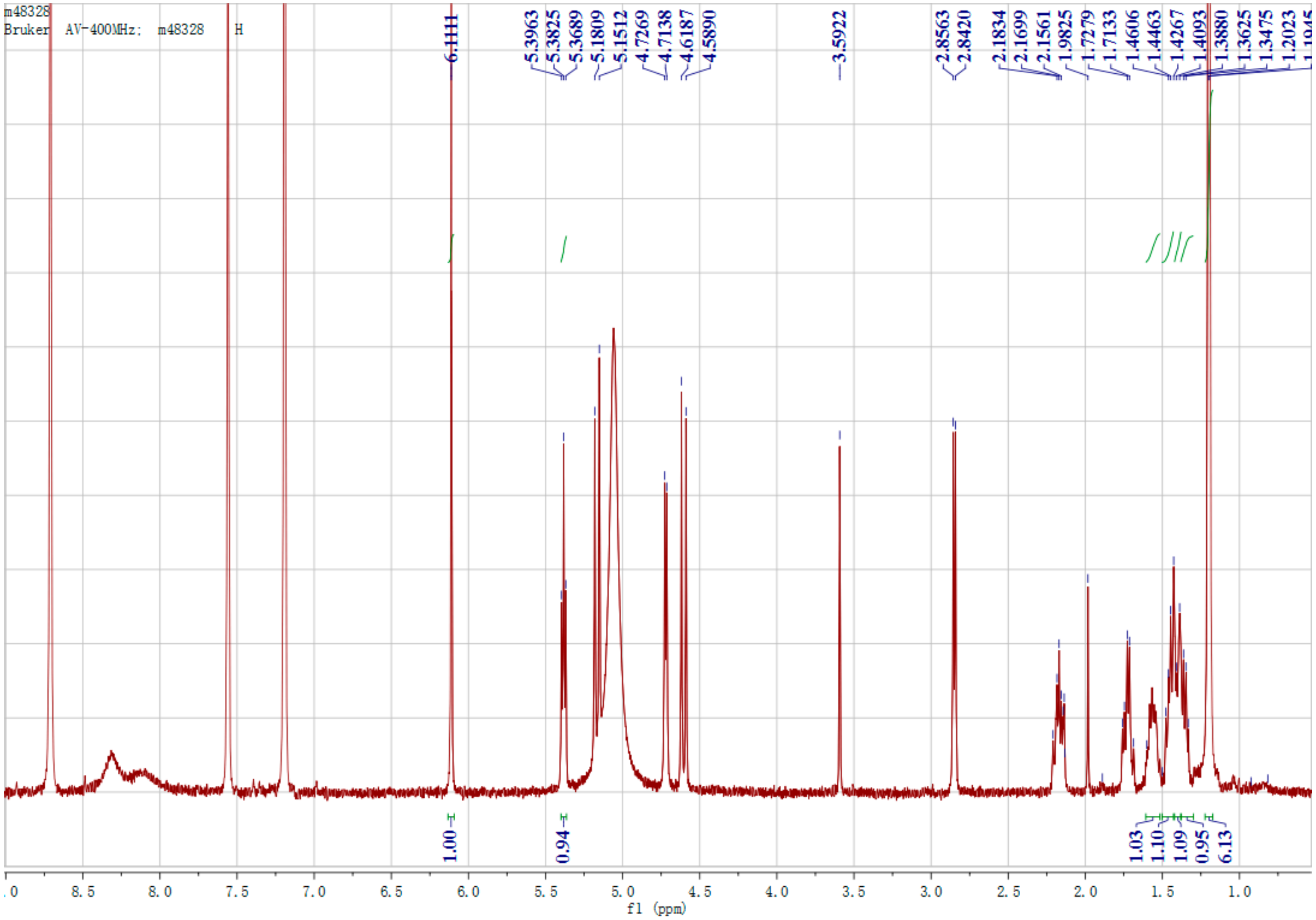

Figure S7. The ^{1}H-NMR of botryosphaerin H in C_5D_5N.

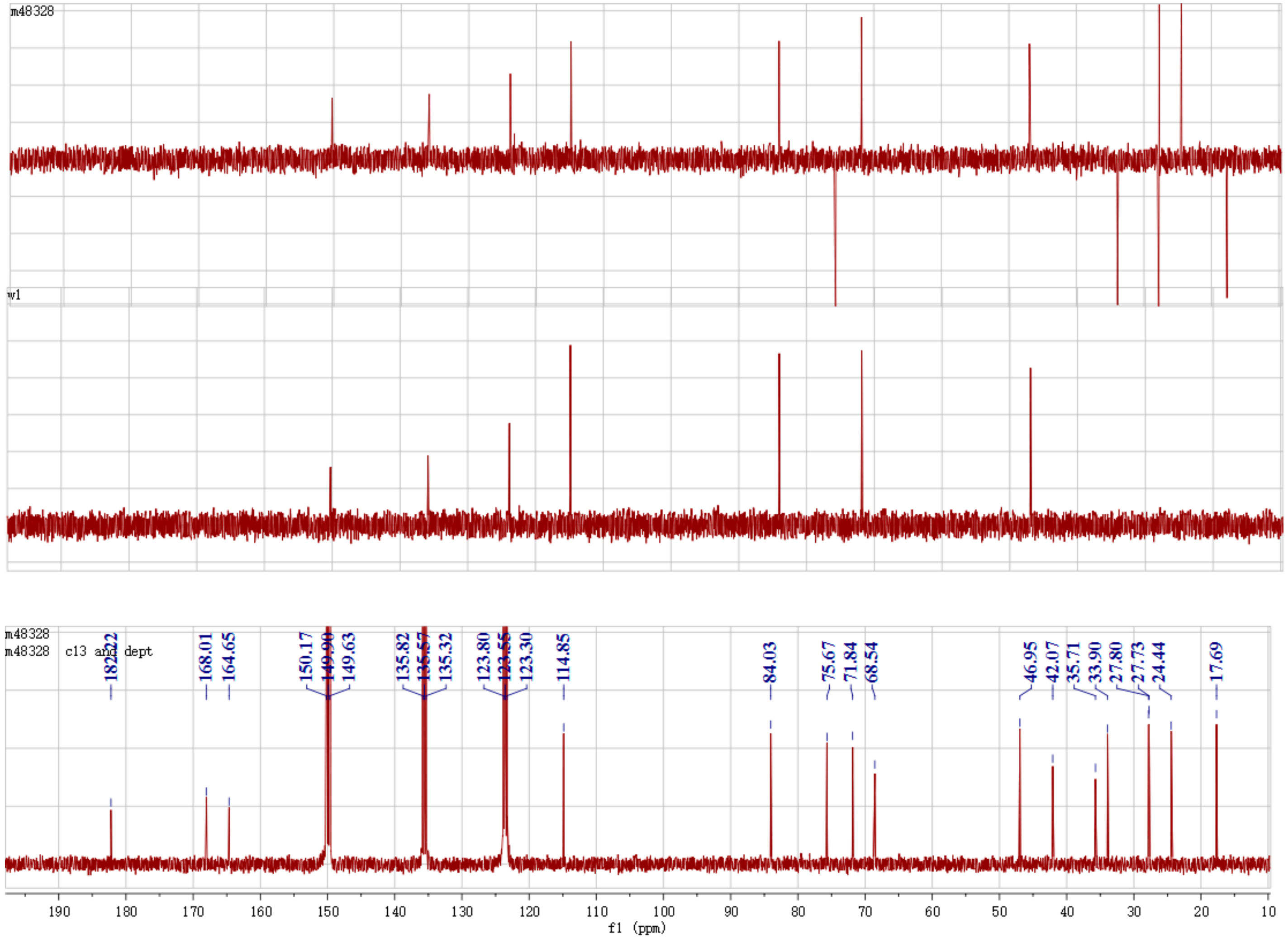

Figure S8. The ^{13}C-NMR of botryosphaerin H in C_5D_5N.

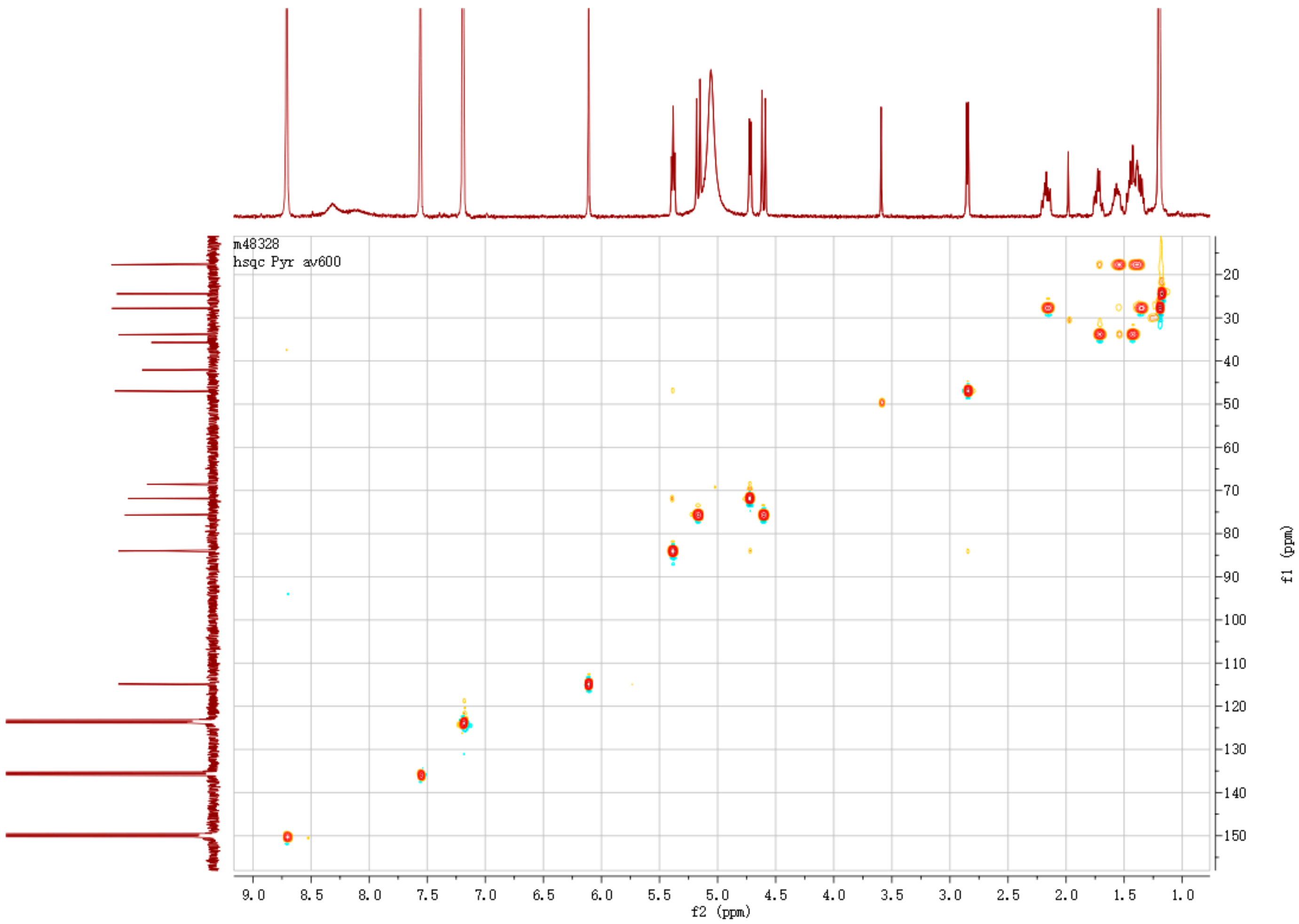

Figure S9. The HSQC NMR of botryosphaerin H in C_5D_5N.

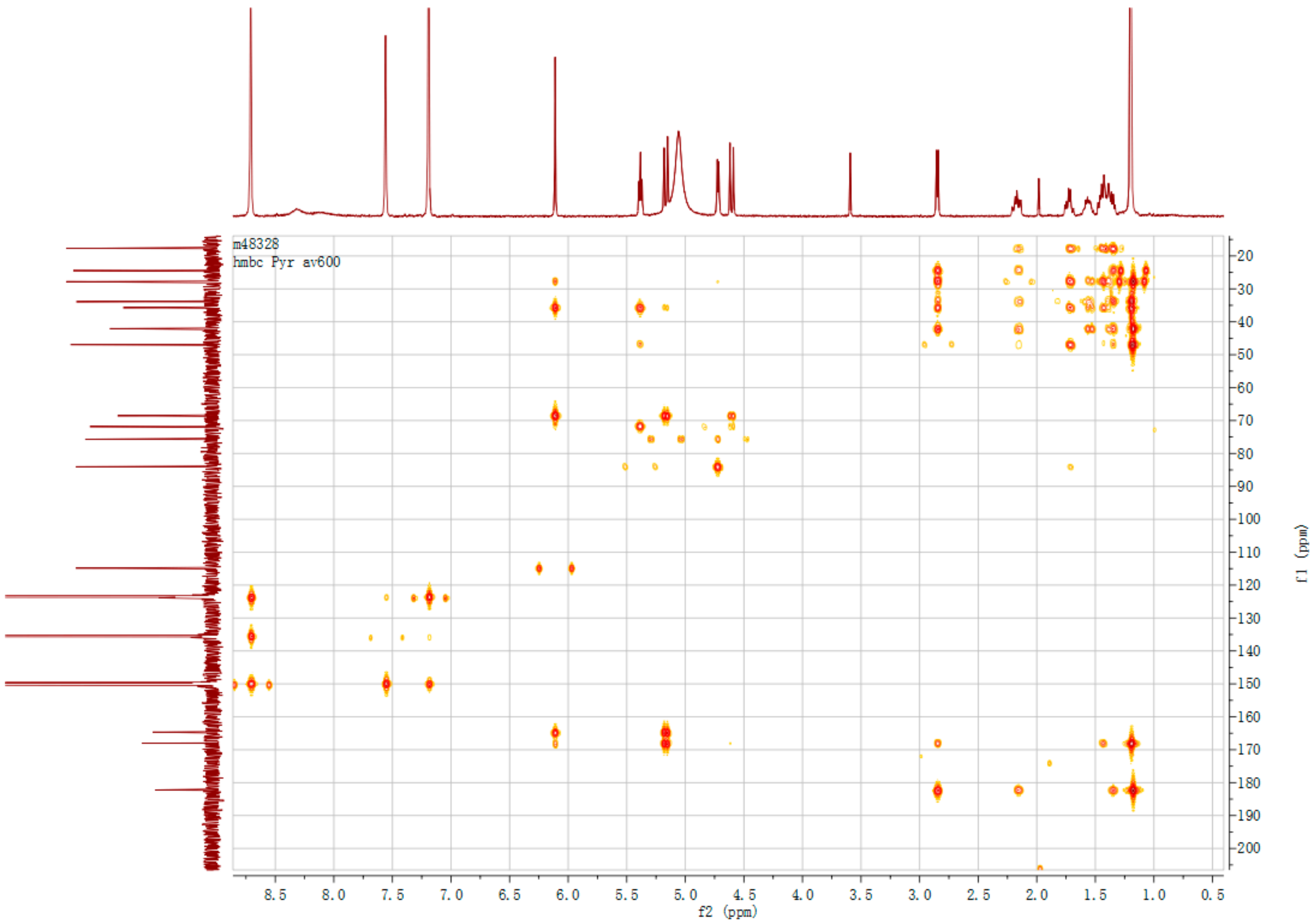

Figure S10. The HMBC NMR of botryosphaerin H in C_5D_5N.

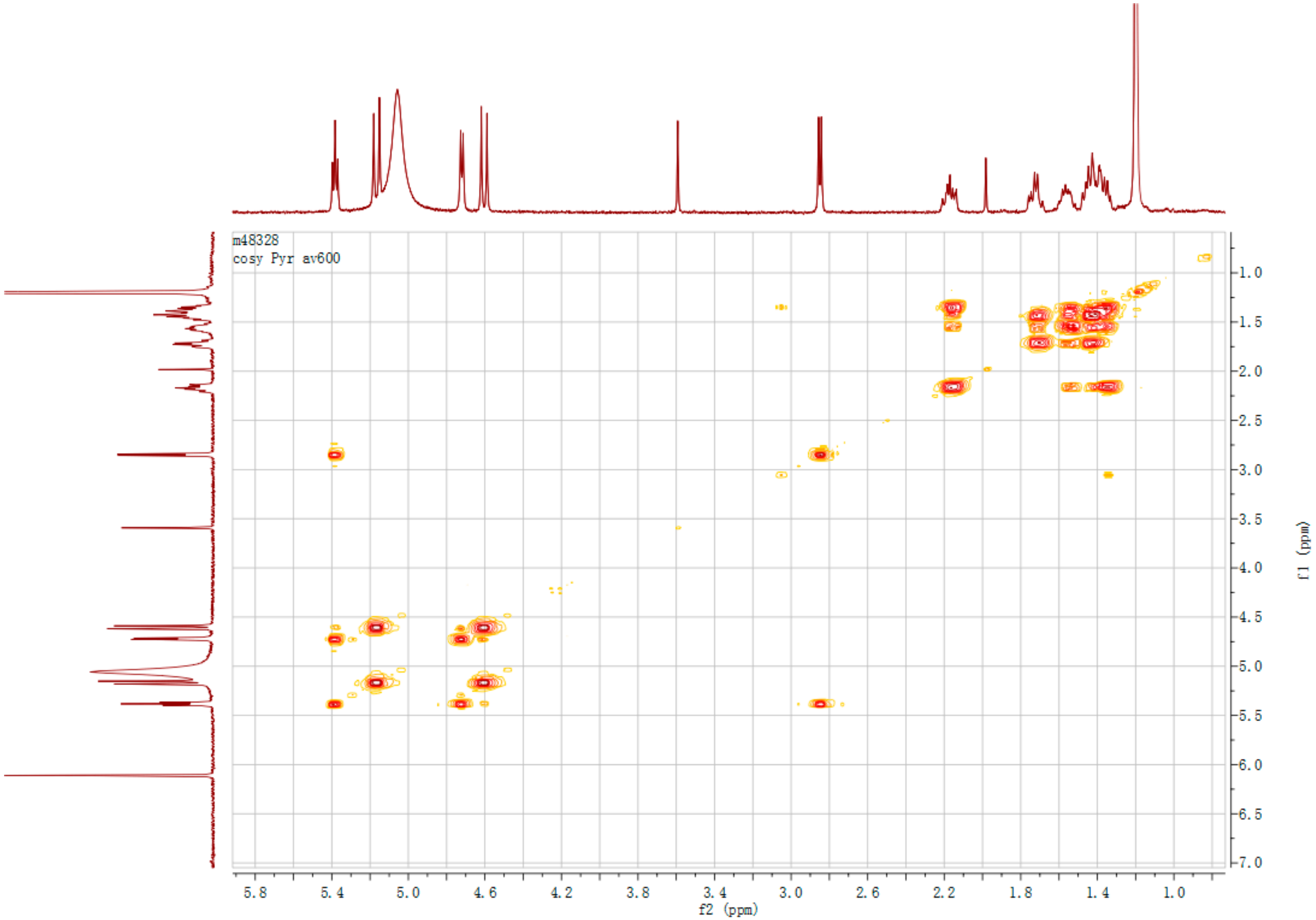

Figure S11. The COSY NMR of botryosphaerin H in C_5D_5N.

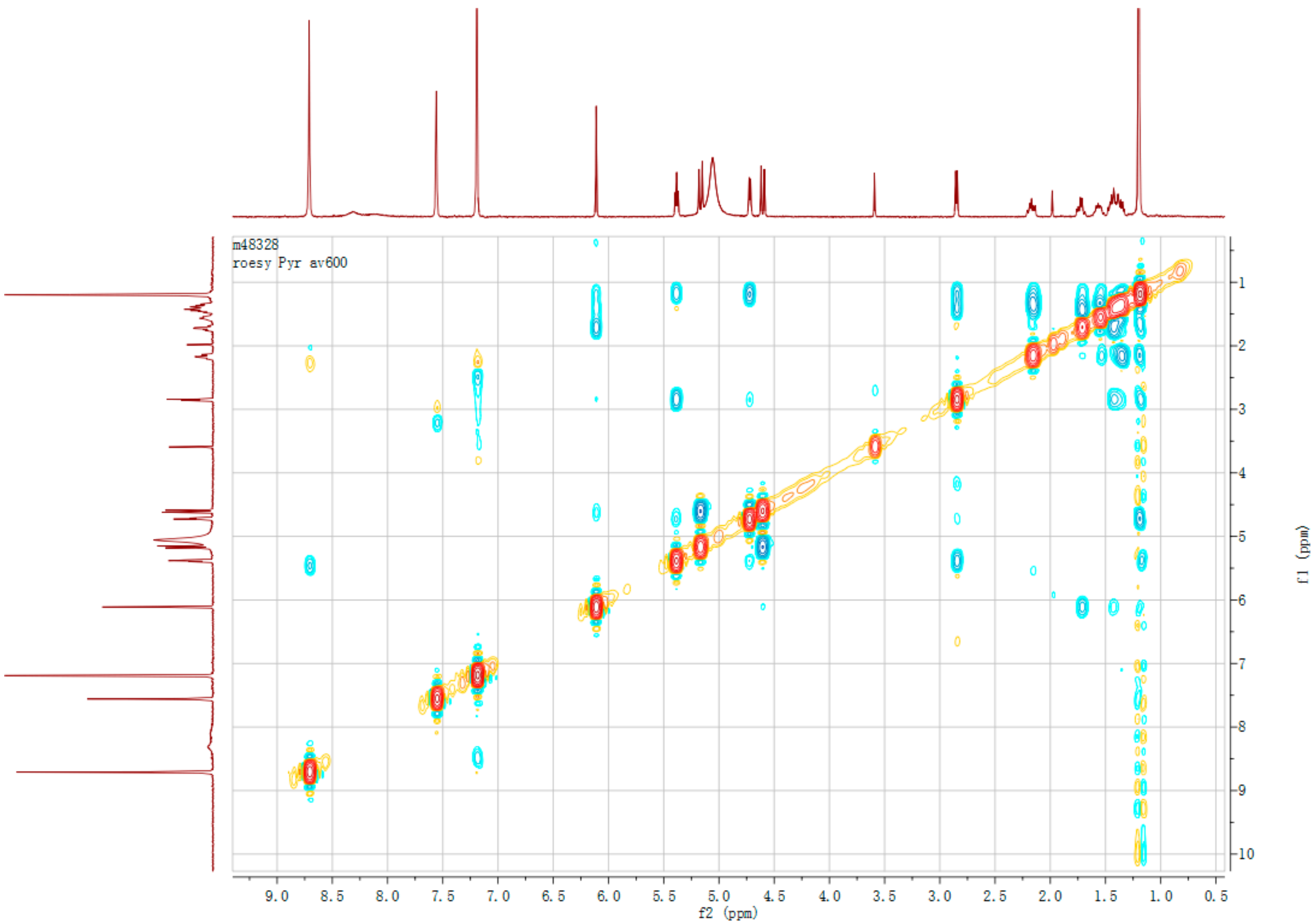

Figure S12. The ROESY NMR of botryosphaerin H in C_5D_5N.
